# Genome Sequence of Alongshan Virus from Ixodes ricinus Ticks Collected in Switzerland

**DOI:** 10.1128/mra.01287-22

**Published:** 2023-02-13

**Authors:** Stefanie Stegmüller, Cornel Fraefel, Jakub Kubacki

**Affiliations:** a Institute of Virology, Vetsuisse Faculty, University of Zurich, Zurich, Switzerland; Queens College Department of Biology

## Abstract

Here, we report the detection of an Alongshan virus (ALSV) strain in Switzerland. Next-generation sequencing of homogenates from Ixodes ricinus ticks collected in Canton Grisons, Switzerland, in 2022 yielded a coding-complete ALSV genome.

## ANNOUNCEMENT

Alongshan virus (ALSV) is a member of the unclassified Jingmenvirus group within the family of the *Flaviviridae*. In contrast to the viruses of the genera Flavivirus, Hepacivirus, Pegivirus, and Pestivirus, which have nonsegmented genomes, the genomes of the unclassified Jingmen viruses are segmented. Specifically, the ALSV genome consists of four segments of positive-sense single-stranded RNA (ssRNA). ALSV was first detected in China in 2017, in patients with a history of tick bites and tick-borne encephalitis virus (TBEV) symptoms but negative TBEV diagnostics (1). In 2019, ALSV was also detected in Ixodes ricinus ticks in Finland, but human infection cases were not found (2).

Our study aimed to determine the virome of ticks collected in 2021 and 2022 from different environments and regions of Switzerland. For sequence analysis, ticks were divided into pools according to species, developmental stage, sex, and place of collection and homogenized in 500 μL of phosphate-buffered saline (PBS) using a TissueLyser II instrument (Qiagen, Germany) at 20 Hz for 2 min. Total RNA was extracted (viral RNA minikit; Qiagen), reverse transcribed (RevertAid first-strand cDNA synthesis kit; Thermo Scientific, USA), and amplified (sequence-independent single-primer amplification [SISPA] protocol), and libraries were prepared for next-generation sequencing (NGS) (NEBNext Ultra II DNA library prep kit; New England BioLabs, USA) as described previously (3). A paired-end NGS run of 2 × 100-nucleotide (nt) read length was performed on an Illumina NovaSeq sequencing system at the Functional Genomics Center Zurich (Switzerland). In total, 3.2 × 10^6^ raw reads were sequenced and quality controlled using FastQC (v0.11.7). PCR primers, sequencing adaptors, and low-quality ends were trimmed by Trimmomatic (v0.39), and contigs were assembled using metaSPAdes (v3.14.0), with settings as described previously (4, 5). Assembled contigs were compared against the NCBI nucleotide database (https://ftp.ncbi.nlm.nih.gov/blast/db/) using blastn (v2.10.1+) and visualized, manually confirmed, and corrected using the SeqMan Pro software v17 (DNAStar; Lasergene, USA) based on available GenBank complete genomes. The coding sequences were determined using open reading frame (ORF) Finder (https://www.ncbi.nlm.nih.gov/orffinder/). Default parameters were used for all software unless otherwise specified.

From a pool of 60 adult male Ixodes ricinus ticks collected in the Canton Grisons, Switzerland, we obtained the coding-complete sequence of segments 1 to 4 of an ALSV genome, with a length of 3,028, 2,788, 2,808, and 2,744 nt; a GC content of 53%, 53%, 54%, and 53%; and a sequencing depth of 3,765×, 4,008×, 3,399×, and 1,909×, respectively. The coding sequences at amino acid levels in blastp analysis (https://blast.ncbi.nlm.nih.gov/) showed high similarities to NS5-like protein (99.8%), glycoprotein VP1a (99.2%), NS3-like protein (99.9%), membrane protein (99.8%), and capsid protein (100%) of an ALSV strain from France (MN095519 to MN095522), and to glycoprotein VP1b (99.6%) of ALSV strains from Finland (MN107154) and Russia (MN648776). This finding expands the list of pathogens that can potentially be transmitted by ticks in Switzerland and warrants further investigations regarding their prevalence in ticks and their impact on public health.

### Data availability.

The generated sequences in this study have been deposited in the NCBI database under GenBank accession numbers OP921096 to OP921099. The raw reads have been deposited under BioProject accession number PRJNA906035 and SRA accession number SRR22427544.
